# Are new national malaria strategic plans informed by the previous ones? A comprehensive assessment of sub-Saharan African countries from 2001 to present

**DOI:** 10.1186/s12936-019-2898-4

**Published:** 2019-07-29

**Authors:** Andrew Andrada, Samantha Herrera, Yazoumé Yé

**Affiliations:** 1 ICF, 530 Gaither Road, Suite 500, Rockville, MD 20850 USA; 2 Save the Children, 899 North Capitol Street NE, #900, Washington, DC 20002 USA

**Keywords:** Malaria, Control, Elimination, Monitoring, Planning, Policy, National malaria strategic plan, Sub-Saharan Africa, Evidence-based

## Abstract

**Background:**

New national malaria strategic plans (NMSPs) should build upon the achievements and challenges identified during the implementation of previous plans, but there is limited research on the transition process between NMSPs. This study aims to fill this gap through an assessment of NMSPs across sub-Saharan Africa.

**Methods:**

The study reviewed the two most recent NMSPs for selected sub-Saharan African countries. Targets for six core malaria indicators were extracted from each NMSP and compared to the coverage achieved according to corresponding population-based surveys completed near the end of the NMSP term. Implementation challenges and proposed solutions identified through the NMSP analysis were documented. The current NMSP was reviewed to determine whether proposed solutions had been integrated into the strategy.

**Results:**

Twenty-two countries in sub-Saharan Africa were included in the assessment. Of the 135 verified targets, only 4 were achieved. No country reached more than one of the six targets assessed in each NMSP. Despite this low success rate, only four of the 22 countries lowered a subsequent target, with most setting the next target at an equal or greater level. Most NMSPs identified solutions to address implementation challenges faced, but the solutions were not always fully incorporated in the new strategy.

**Conclusions:**

The results show a disconnect between NMSPs. Most targets were set according to global goals rather than the individual country’s previous achievements and limitations. This indicates a need to revise the NMSP development process to guide programmes in defining targets based on their country context and incorporate strategies to address challenges identified in the previous NMSP. This will allow countries to set and meet achievable targets as they work toward global goals.

**Electronic supplementary material:**

The online version of this article (10.1186/s12936-019-2898-4) contains supplementary material, which is available to authorized users.

## Background

In 1998, there were nearly 300 million cases of malaria worldwide [1]. The malaria burden led to a renewed focus on malaria control, and the World Health Organization (WHO) and its partners established the Roll Back Malaria (RBM) Partnership in 1998 to harmonize global efforts in the fight against malaria [2]. Sub-Saharan Africa carried the heaviest burden, accounting for 80% of the global malaria deaths [1]. This prompted African heads of state to agree to a common goal of halving Africa’s malaria mortality through the strategies and malaria control targets set by RBM in the Abuja Declaration [3]. Since the Abuja Declaration, the RBM Partnership has worked with national malaria control programmes (NMCPs) of malaria endemic countries to develop their national malaria strategic plans (NMSPs) with the goal of reducing their malaria burden and deaths due to malaria [4]. The NMSP describes a country’s approach to reducing the malaria burden and details the interventions, areas of focus, timeline, and necessary budget [4]. Having a standardized plan allows countries to follow a road map and measure their progress throughout different iterations as they work toward internationally agreed-upon goals.

Adequate transition from one NMSP to the next is essential for continuous improvement of malaria control efforts, and informing a new NMSP based on the achievements of the previous plan is vital. As a country’s NMSP term nears its end, RBM and its partners implement a malaria programme review (MPR) to assess programme management, identify challenges, and develop strategies to improve the next NMSP [5]. The challenges and action points enumerated in the review are helpful for informing NMSP development. Despite this review process, there is limited documentation and research on the transition between one plan and the next and MPR findings are not always incorporated into the next NMSP.

This study aims to fill this gap through a comprehensive assessment of NMSPs across sub-Saharan Africa. The objectives include a review of individual country achievements among selected NMSP targets and a documentation of the challenges identified in previous implementations and the solutions proposed in the current NMSP.

## Methods

### Study design and scope

This study consisted of a desk review of NMSPs and population-based surveys available from selected malaria endemic countries in sub-Saharan Africa. The review focused on the two most recent NMSPs and corresponding population-based surveys for each country because of their availability and insight into current implementation challenges and solutions. Twenty-two countries were included in the assessment, representing 82% of the global malaria burden [6]. These countries provide a diverse background of malaria transmission settings, support from donors, and maturity of NMCPs for review of different target setting, coverage achievements, challenges, and solutions.

### Data sources

The primary data sources were NMSPs, Demographic and Health Surveys (DHS), Malaria Indicator Surveys (MIS), and Multiple Indicator Cluster Surveys (MICS). MPR reports and national malaria monitoring and evaluation (M&E) plans were reviewed for additional information. These documents were obtained through primary sources (e.g., NMCP websites and contact persons) and secondary sources (e.g., RBM and online searches).

#### NMSP, including monitoring and evaluation plan

The NMSP outlines a country’s malaria prevention and treatment strategies. The plan includes a country’s profile, malaria situation analysis, strategic plan, implementation strategies and M&E plan. The M&E plan provides a framework for tracking the implementation of the NMSP and describes indicators, data sources, frequency of data collection, data analysis, data dissemination and data use to inform programme performance.

#### MPR

The MPR provides a comprehensive mixed-methods review of the NMCP. An MPR is led by the NMCP in collaboration with the WHO and its partners. The review is conducted during the mid-term and toward the end of the NMSP lifecycle, prior to the development of a new NMSP. The results are presented by thematic areas to assist the NMCP in addressing weaknesses to help the programme reach its goals [5].

#### Population-based surveys

Population-based surveys collect and present information on monitoring and impact indicators. The DHS Program, together with countries worldwide, conduct nationally representative surveys that cover health topics, such as malaria [7]. In comparison, MICS is a household survey implemented by government organizations in collaboration with UNICEF that generates data on indicators focused on children and women [8]. The MIS is specific to malaria, with methodology developed by the RBM Monitoring and Evaluation Reference Group (MERG), and is implemented by various organizations [9].

### Data extraction

Six core malaria indicators were extracted for comparison across each NMSP. These indicators include: (1) proportion of households owning at least one insecticide-treated net (ITN); (2) proportion of children under five who slept under an ITN the previous night; (3) proportion of pregnant women who slept under an ITN the previous night; (4) proportion of pregnant women receiving intermittent preventive treatment in pregnancy (IPTp) during an antenatal care (ANC) visit; (5) proportion of children under five with fever who had a finger or heel stick; and (6) proportion of children with fever who received any anti-malarial drugs. These core indicators encompass important malaria interventions across vector control and case management and are recommended by the RBM MERG as key indicators for assessing malaria control programmes for prevention and treatment [10]. These indicators are available for most countries through population-based surveys. The targets for each of these indicators were extracted from both NMSPs reviewed for each country.

NMSPs provide the NMCP’s analysis of the previous strategic plan. This analysis was examined for implementation challenges, which were extracted and categorized by specific malaria control activities. Any solutions provided for each challenge were also extracted. The subsequent plan was reviewed for further solutions and strategies that would improve on the previous challenges identified. All the data was extracted into Microsoft Excel. The first author reviewed all relevant documents and extracted all the data. The senior author reviewed all targets, coverage achieved, challenges and solutions. Any discrepancies in the data were rectified prior to the synthesis. A final review was conducted by the first author.

### Data synthesis

To assess each country’s achievements, relevant data sources within the NMSP’s timeframe were reviewed. Population-based surveys conducted closest to the NMSP term end were used to provide actual coverage achieved. Differences between the first NMSP targets for each indicator and actual coverage were calculated. The first NMSP targets were also compared to the subsequent NMSP targets to assess any changes in target setting. To determine the effects on strategic development, the implementation challenges indicated in the NMSP and MPR were reviewed across malaria control activities, which included integrated vector management (IVM); malaria in pregnancy; case management; social and behaviour change communication (SBCC); and surveillance, monitoring, and evaluation (SME). The solutions were qualitatively assessed to determine whether they fully addressed each challenge, and the subsequent NMSP strategy was reviewed to assess whether the proposed solutions were incorporated. Each challenge extracted was categorized by theme (e.g., funding, capacity, and SME). Common challenges that were not addressed were documented.

## Results

### Sample description

**Fig. 1:**
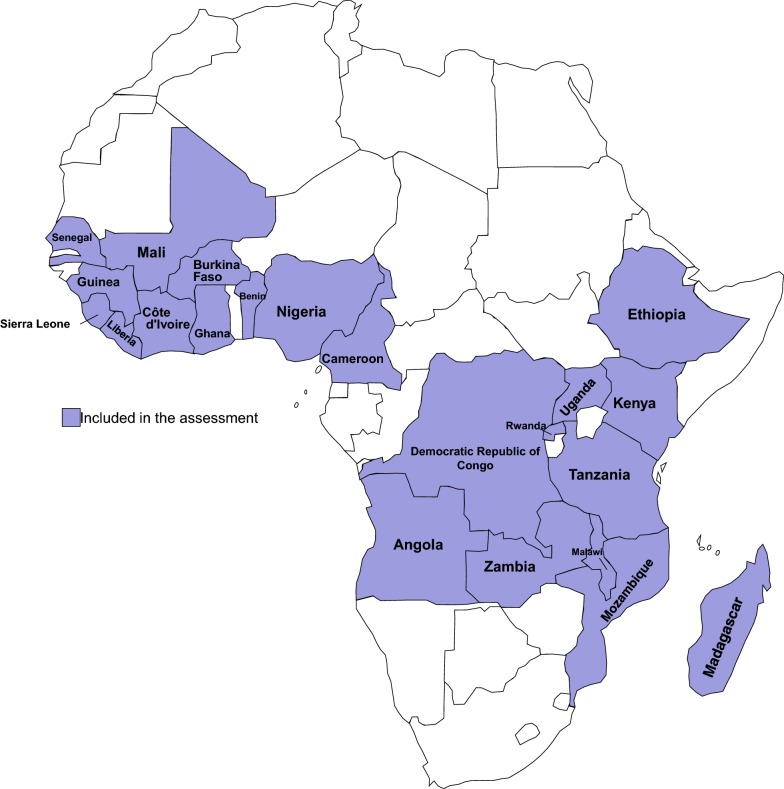
Map of countries included in the assessment

NMSPs from 22 countries in sub-Saharan Africa were included in the assessment (Fig. 1). For each country, the current strategic plan and its predecessor were reviewed, for a total of 44 NMSPs (Table 1). The earliest strategic plan began in 2001 from Kenya and the most recent plans continued until 2022 from Madagascar, Malawi and Mozambique. Fourteen MIS, six DHS and two MICS surveys were reviewed to determine actual coverage achieved.

**Table 1:** NMSP and population-based surveys

Country	NMSP	Population-based survey	Estimated malaria cases^a^ [6]
Angola	2008–2013	DHS 2015–2016	4,615,605
	2016–2020		
Benin	2011–2015	MIS 2015	4,111,699
	2017–2021		
Burkina Faso	2011–2015	MIS 2014	7,907,562
	2016–2020		
Cameroon	2011–2015	MICS 2014	7,307,515
	2014–2018		
Côte d’Ivoire	2012–2015	MICS 2016	3,373,486
	2016–2020		
DRC	2013–2015	DHS 2013–2014	25,021,891
	2016–2020		
Ethiopia	2011–2015	MIS 2015	2,666,954
	2017–2020		
Ghana	2008–2015	MIS 2016	7,805,045
	2014–2020		
Guinea	2006–2010	DHS 2012	4,282,165
	2013–2017		
Kenya	2001–2010	MIS 2010	3,520,384
	2009–2018		
Liberia	2010–2015	MIS 2016	911,333
	2016–2020		
Madagascar	2013–2017	MIS 2016	2,324,289
	2018–2022		
Malawi	2011–2015	DHS 2015–2016	4,303,543
	2017–2022		
Mali	2013–2017	MIS 2015	7,160,192
	2016–2018		
Mozambique	2012–2016	MIS 2015	10,025,823
	2017–2022		
Nigeria	2009–2013	DHS 2013	53,667,565
	2014–2020		
Rwanda	2008–2012	MIS 2013	6,172,220
	2013–2020		
Senegal	2011–2015	DHS 2015	1,024,285
	2016–2020		
Sierra Leone	2011–2015	MIS 2016	2,869,588
	2016–2020		
Tanzania	2008–2013	HMIS 2011–2012	6,477,825
	2014–2020		
Uganda	2010–2015	MIS 2014–2015	8,600,724
	2014–2020		
Zambia	2011–2015	MIS 2015	3,475,522
	2017–2021		

All indicators were reviewed from the Tanzania DHS 2015–2016 and no targets were met

^a^There was an estimated 219,000,000 malaria cases globally in 2017 [6]

### Target setting and achievement

#### Vector control

##### Household ownership of at least one ITN

**Fig. 2:**
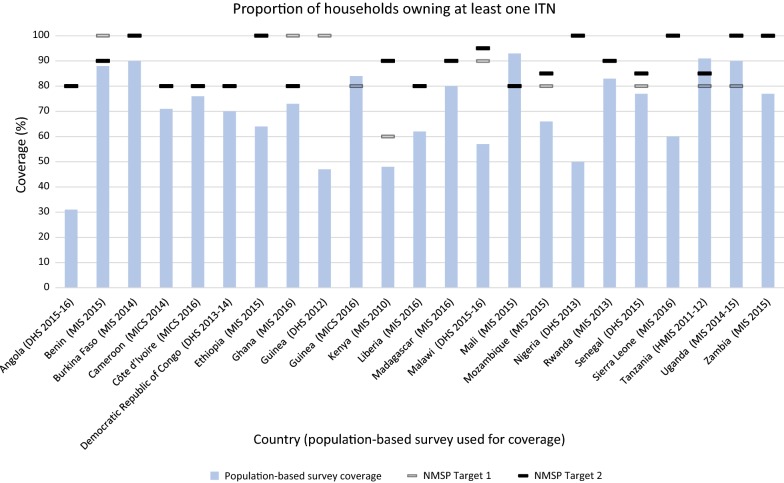
Proportion of households owning at least one ITN. *ITN* insecticide-treated net. NMSP Target 1 = first NMSP reviewed, NMSP Target 2 = subsequent NMSP reviewed. Countries that did not change their target may be indicated with one line

All 44 NMSPs indicated a target for the indicator for household ownership of at least one ITN. The targets ranged from 60 to 100% coverage, with the largest proportion of targets (40.9%) set at 80% coverage (Fig. 2). The gap between the NMSP target and coverage measured through population-based surveys ranged from 53% below target to 13% above target. Four of the 22 countries surpassed their household ITN ownership target (Guinea, Mali, Tanzania, and Uganda). Despite not achieving their set target, 19 countries kept their target the same or increased it in the subsequent plan. Benin, Ghana and Guinea were the only countries to decrease their target. Benin decreased the target from 100% in the 2011–2015 NMSP to 90% in the 2017–2021 NMSP, Ghana decreased the target from 100% in the 2008–2015 NMSP to 80% in the 2014–2020 NMSP and Guinea decreased the target from 100% in the 2006–2010 NMSP to 80% in the 2013–2017 NMSP.

##### ITN use among children under five

**Fig. 3:**
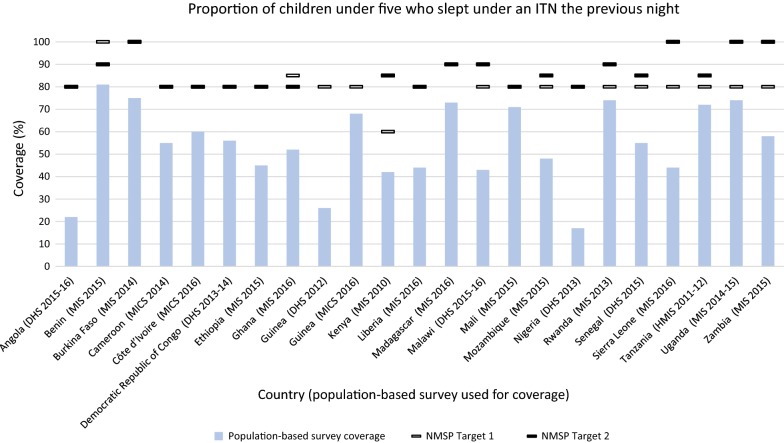
Proportion of children under five who slept under an ITN the previous night. *ITN* insecticide-treated net. NMSP Target 1 = first NMSP reviewed, NMSP Target 2 = subsequent NMSP reviewed. Countries that did not change their target may be indicated with one line

Each NMSP indicated a target for the proportion of children under five who slept under an ITN the previous night. The targets ranged from 60 to 100% coverage, with a majority of targets (72.7%) set at 80% coverage (Fig. 3). No countries were able to meet their target. The gap between target and measured coverage ranged from 63 to 6% below target. The largest gap was observed in Nigeria. Mali, Rwanda, Tanzania and Uganda achieved within 10% of their target. Benin and Ghana were the only countries to decrease their target in the subsequent NMSP. Benin decreased the target from 100% in the 2011–2015 NMSP to 90% in the 2017–2021 NMSP, and Ghana decreased the target from 85% in the 2008–2015 NMSP to 80% in the 2014–2020 NMSP.

##### ITN use among pregnant women

**Fig. 4:**
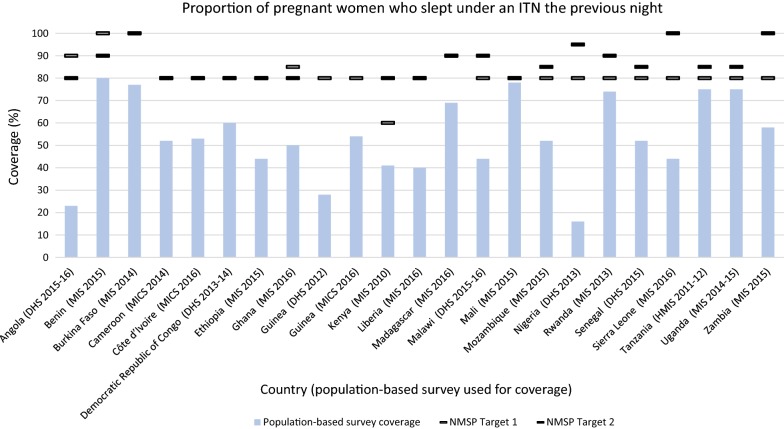
Proportion of pregnant women who slept under an ITN the previous night. *ITN* insecticide-treated net. NMSP Target 1 = first NMSP reviewed, NMSP Target 2 = subsequent NMSP reviewed. Countries that did not change their target may be indicated with one line

Each NMSP indicated a target for the proportion of pregnant women who slept under an ITN the previous night. The targets ranged from 60 to 100% coverage, with a majority of targets (68.8%) set at 80% coverage (Fig. 4). No countries were able to meet their target. The measured coverage was similar to the ITN use among children under five indicator and ranged from 67 to 2% below target. The largest gap was observed in Angola. Mali, Rwanda, Tanzania and Uganda achieved within 10% of their target. Angola, Benin, and Ghana decreased their targets in the subsequent NMSP. Angola decreased the target from 90% in the 2008–2013 NMSP to 80% in the 2016–2020 NMSP. Benin decreased the target from 100% in the 2011–2015 NMSP to 90% in the 2017–2021 NMSP, and Ghana decreased the target from 85% in the 2008–2015 NMSP to 80% in the 2014–2020 NMSP.

##### Pregnant women receiving IPTp during an ANC visit

**Fig. 5:**
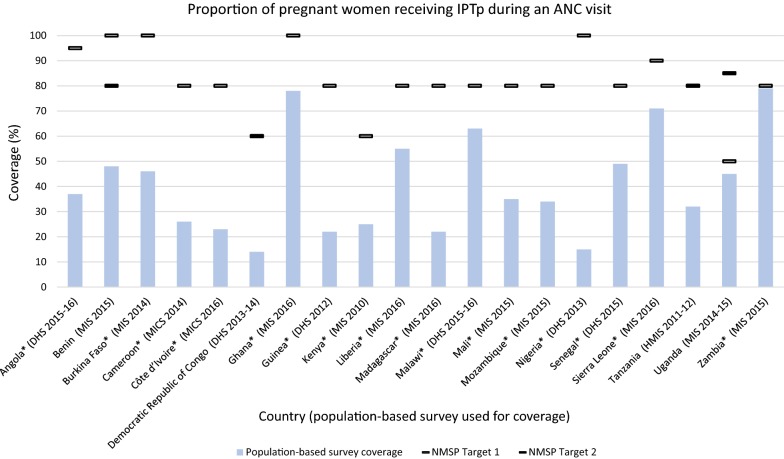
Proportion of pregnant women receiving IPTp during an ANC visit. *IPTp* intermittent preventative treatment in pregnancy, *ANC* antenatal care.*Indicates countries that shifted their first NMSP indicator of IPTp2 to IPTp3 in the subsequent NMSP. NMSP Target 1 = first NMSP reviewed, NMSP Target 2 = subsequent NMSP reviewed. Results show IPTp2 targets and coverage achieved

Forty of the 44 NMSPs indicated targets for pregnant women receiving IPTp during an ANC visit. Rwanda and Ethiopia did not include IPTp as a malaria intervention, citing concerns of sulfadoxine–pyrimethamine resistance. The targets ranged from 50 to 100% coverage with the majority of targets (55%) at 80% coverage (Fig. 5). According to the corresponding surveys, no countries met their targets. The gap between coverage and target ranged from 85 to 1% below target. The largest gap was observed in Nigeria. Uganda and Zambia achieved coverage within 5% of the target. Sixteen countries updated their IPTp indicator from two doses of IPTp to three or more doses. Benin and DRC included included targets for both. Malawi set their IPTp3+ target at 60% and the other countries making this shift, set targets at 80% or above. Tanzania and Uganda kept their targets at two or more doses of IPTp, with Tanzania keeping the same target of 80% and Uganda increasing their 2010–2015 NMSP target of 50% to 85% in the 2014–2020 NMSP.

#### Case management

##### Malaria diagnostic test among children under five

**Fig. 6:**
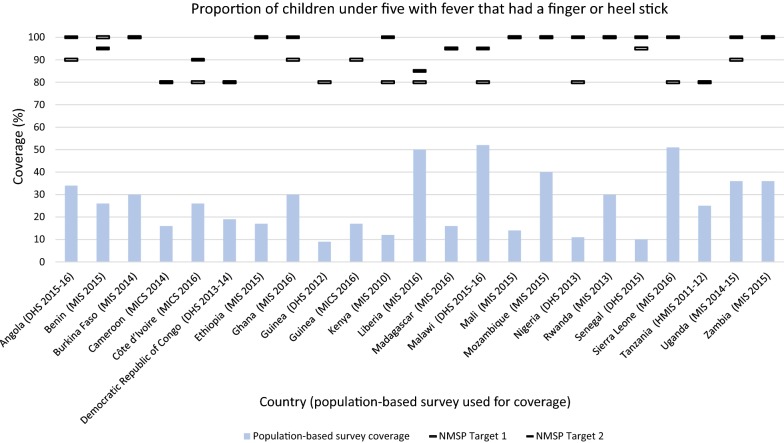
Proportion of children under five with fever that had a finger or heel stick. NMSP Target 1 = first NMSP reviewed, NMSP Target 2 = subsequent NMSP reviewed. Countries that did not change their target may be indicated with one line

Each NMSP indicated a target for children under five with fever in the previous 2 weeks who had a finger or heel stick. The targets ranged from 80 to 100%, with the largest proportion (45.4%) at 80% coverage (Fig. 6). No country achieved its target coverage. Significant gaps were observed between the NMSP and the corresponding population-based survey, with a range of 86% to 28% below target. The largest gap was observed in Mali. Malawi, Liberia, and Sierra Leone achieved coverage within 30% of their target. Benin was the only country to lower its target, decreasing the 2011–2015 NMSP target from 100 to 95% in the 2017–2021 NMSP.

#### Use of anti-malarial among children under five with fever

**Fig. 7:**
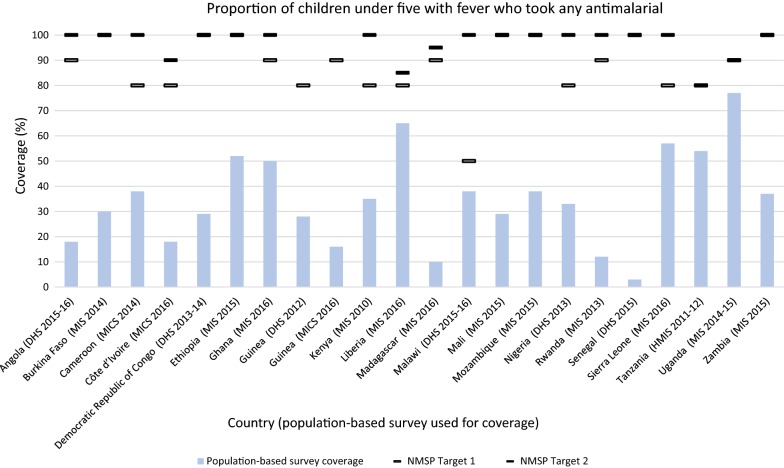
Proportion of children under five with fever who took any antimalarial. NMSP Target 1 = first NMSP reviewed, NMSP Target 2 = subsequent NMSP reviewed. Countries that did not change their target may be indicated with one line

Each NMSP indicated a target for children with fever receiving an anti-malarial. The targets ranged from 50 to 100%, with most countries indicating either 80% or 100% coverage (Fig. 7). No country reached its target coverage. The gap between the NMSP target and coverage measured through the corresponding population-based surveys ranged from 97% below target to 8% below target. Significant gaps were observed in Madagascar, Rwanda, and Senegal. Malawi was the only country to achieve coverage within 10% of its target. Benin was the sole country to lower their target in the subsequent NMSP, decreasing their 2011–2015 NMSP target from 100 to 90% in the 2017–2021 NMSP.

##### NMSP implementation challenges and proposed solutions

Twenty-one of the 22 NMSPs examined provided an analysis of the previous NMSP implementation, with most informed by a recent MPR. The implementation challenges included issues with funding, lack of coordination within the Ministry of Health (MoH) and a lack of coordination between NMCPs and the private sector and general staff capacity (Table 2). A complete table of extracted challenges and solutions for each country may be found in Additional file 1: Table S1.

**Table 2:** Implementation challenges and solutions

Category	Implementation challenge	Solution
Funding	Insufficient funding for IVM activities (indoor residual spraying, larval source management, and ITN mass distribution campaigns), diagnostic tools, first-line treatments and sulfadoxine–pyrimethamine for IPTp, and SBCC activities	Mobilize resources in the government through the elevation of the NMCP within the ministry Advocate more government funding and seek additional donors
Planning and coordination	Absence of an exit strategy for free distribution of ITNs No plan for sustainability of routine ITN distribution SBCC plans without clear objectives Weak coordination and integration across the ministry Poor management of supply chain systems	Improve ITN distribution strategies through campaigns and routine health services Increase collaboration with the private sector and across other ministries Establishing an integrated SBCC plan
Staff capacity	Lack of coordination across organizations implementing SBCC activities Low staff capacity to conduct malaria control activities and limited experience with IVM Training needed for health facility staff to make a diagnosis Lack of entomological experience	Increase capacity of health facility staff and train community health workers
SME	Weak HIS Lack of integration of monitoring systems Delayed reporting and neglected malaria indicators Insufficient SME capacity to conduct analysis and interpret data Incomplete data collection and poor data quality Inadequate data management and use	Strengthen HIS systems Increase data use and implement quarterly reports Improved communication within SME unit

#### Insufficient funding

The most commonly cited implementation challenge across all malaria control activities was insufficient funding. This underfunding resulted from inadequate resources received from donors, a small government fund allocation, or an improper budget estimation. Without these resources, programmes indicated that they were unable to implement planned IVM activities such as indoor residual spraying, larval source management or ITN mass distribution campaigns. Malaria in pregnancy and case management plans were also affected, leading to limited amounts of first-line treatments, rapid diagnostic tests and medicines for IPTp. In addition, programmes were unable to execute their planned SBCC activities to help raise public awareness of these interventions. The NMSPs cited a plan to mobilize resources within the government through the elevation of the NMCP within the MoH, advocate more government funding and seek additional donors. Funding was a challenge that countries did not always integrate a solution into their next plan.

#### Inadequate planning and coordination

Another common challenge indicated in the NMSPs was inadequate planning of IVM activities and poor inventory management. Inadequate planning resulted in the absence of an exit strategy for free distribution of ITNs and the lack of a plan for the sustainability of routine ITN distribution, resulting in areas without access to ITNs. This lack of preparation extended to SBCC plans with no clear objectives and led to difficulty in launching activities. NMSPs also noted poor management of supply chain systems, which resulted in stockouts of medicines, rapid diagnostic tests, and microscopes for diagnosis.

The NMSPs described the obstacle of weak coordination and integration across the ministry and within relevant sectors in the MoH. Other ministries include the Ministry of Agriculture and Irrigation, to coordinate the prevention of vector proliferation and disease transmission. Relevant sectors in the MoH included the Reproductive Health division, to prevent malaria in pregnancy and increase access to prevention methods and treatment. Coordination challenges extended to the private sector and decentralized communities, which led to unavailability of ITNs and treatments. NMSPs also reported a lack of coordination across organizations implementing SBCC activities to avoid conflicting behaviour change messages.

Solutions detailed across NMSPs included developing an improved strategy for ITN distribution through campaigns and routine health services, increasing collaboration with the private sector and across other ministries, establishing an integrated SBCC plan, and expanding communication for SBCC and social mobilization. A lack of coordination across the MoH or private sector was a common challenge without a solution incorporated into the next NMSP.

#### Low capacity of staff

NMSPs specified a lack of overall technical capacity to conduct malaria control activities, which include a lack of entomological expertise and tools for effective malaria vector monitoring and surveillance. This inexperience restricted NMCP’s ability to conduct entomological surveillance and interpret data to guide IVM. It also affected the execution of IVM activities, such as larval source management, indoor residual spraying, and ITN mass distribution. The lack of entomological surveillance data lead to some NMCPs to misallocate ITNs and prohibited the development of strategies against insecticide resistance. These capacity issues extended to additional training needed for health facility staff to properly conduct diagnostic tests to confirm suspected malaria cases and to supervise provision of IPTp. Other staff capacity issues included insufficient SME staff capacity to analyse and interpret data, leading to a lack of quarterly review.

Issues pertaining to building capacity of staff were often addressed in the next NMSP through trainings of health facility staff and community health workers. Countries citing capacity challenges in entomology or SME did not always indicate a solution in their subsequent NMSP.

#### SME

SME challenges commonly noted in NMSPs included weak health information systems (HIS), incomplete data collection, poor data quality, inadequate data management and use and a lack of integration of monitoring systems. Data management issues included delays in reporting and neglected malaria indicators. These issues led to NMCPs inability to determine coverage or quantify the needs of malaria prevention and control interventions.

To resolve SME issues, NMSPs indicated plans to strengthen health management information systems, increase data use and implement the publication of quarterly reports. They also proposed better coordination within the SME unit through supervisory roles and development of comprehensive M&E plans. Data management issues were not always accounted for in the subsequent NMSP.

## Discussion

This study examined the achievements of select sub-Saharan African countries toward their NMSP targets and documented previous NMSP implementation challenges and solutions proposed in the current NMSP. Despite unsuccessfully achieving their targets, most countries continued to set their NMSP targets at an equal or higher value. Each country experienced implementation challenges but had difficulty incorporating solutions into new plans due to the complexity of those challenges.

Household ownership of at least one ITN was the only target achieved out of the six indicators examined, with large gaps observed among the other five indicators. Achievement of this target is largely due to the increased international donor support since 2003, which totaled $1.6 billion by 2011 [11, 12]. With these funds and direction from RBM, programmes have focused on the rapid, national scale-up of ITN distribution [13]. ITN mass distribution has been demonstrated to rapidly increase ITN ownership, and this simultaneous rollout by endemic countries is consistent with an overall increase in ITN ownership across sub-Saharan Africa [14, 15]. This rapid increase in ITN ownership, however, has not necessarily translated into increased ITN use. No countries in this study met their targets for ITN use among children and pregnant women. Implementation challenges, such as insufficient funding and a lack of planning for SBCC activities, which have been effective in increasing ITN use across sub-Saharan Africa [16, 17], may have contributed to countries falling short of their targets [18–20]. Other possible factors for low ITN use include inadequate access to nets, insufficient amount of nets, lack of replacement strategy and education level of the household head [21–24].

The largest gaps between coverage and targets were observed across the IPTp, malaria diagnosis, and treatment indicators. Several health system barriers, such as poor leadership, low funding allocation, and human resource challenges, have led to lower IPTp coverage [25]. The stockout of supplies and non-adherence to treatment guidelines was a commonly cited challenge. This may have resulted in the gap in coverage and low-quality malaria case management, which has been shown to affect countries in sub-Saharan Africa [26, 27]. To address these issues, NMCPs have modified their subsequent strategy to incorporate malaria diagnosis in their integrated community case management to increase access and improve case management [28]. Further integration of malaria services into maternal and child health programmes were also listed as solutions to provide pregnant women with ITNs and IPTp as a strategy to reduce malaria in pregnancy.

Despite the gaps between the NMSP targets set and what was achieved, 18 of the 22 countries kept the same target or increased it for the next NMSP. The target setting appeared to follow the internationally agreed-upon goals set for Africa by WHO and RBM and its partners and the Millennium Development Goals and Sustainable Development Goals (SDGs) [29, 30]. NMSP targets were set at 60% for most indicators from 2000 to 2005, matching the Abuja Declaration targets [3]. Plans developed after 2005 had increased targets to 80% or above, aimed toward RBM’s Global Strategic Plan 2005–2010 and the Global Malaria Action Plan, which had the goal of scaling up malaria control interventions for impact [31, 32]. Among the four countries that lowered their targets, each target was already set above these internationally agreed-upon goals and was lowered to the same level as the international goals, although there was no indication as to why the targets were lowered. In addition, countries updated their malaria prevention in pregnancy strategies and increased the IPTp indicator from two doses to three or more, aligned with updated WHO IPTp guidance [33].

This adherence to international goals regardless of previous accomplishments is rooted in the complex issues that NMCPs face during the NMSP development process. The predominant implementation challenges faced include finances, coordination, staff availability and skill, and SME, each of which was never fully addressed in subsequent NMSPs. NMCPs face the challenge of setting ambitious targets to procure necessary funding for all activities. Lowering their targets may lead to less donor funding. Despite the dramatic increase in funding received by NMCPs, the majority of that funding has come from international aid [34]. Governments from endemic countries made up only 31% of the total funding toward malaria control programmes in Africa, leaving a large gap in necessary funding to scale up interventions and sustain malaria control [35, 36]. According to the WHO Global Technical Strategy for Malaria, an additional $6.4 billion is needed to reach the goal of a 40% reduction in malaria incidence and mortality by 2020 [37]. In addition, $10.1 billion is required to implement NMSPs across 30 African countries from 2018 to 2020, with $4.7 billion yet to be financed [38]. Elevating the NMCP within the ministry and advocating more resources from the local government may help close the gap and sustain malaria control activities, but there is still a heavy reliance on donors. As donor support decreases over time, programmes are uncertain about future funding, making it difficult to incorporate financial solutions in subsequent NMSPs [39]. This lack of funding, coupled with inexperience conducting malaria control activities, contributes to a country’s capacity issues. NMCPs are constrained in their ability to hire and train more staff, which may undermine what they intend to accomplish.

Countries develop malaria M&E plans to track their progress in the fight against malaria. According to the implementation challenges identified, many countries face issues of a weak HIS, poor data management, and insufficient capacity. The insufficient use of data may contribute to the disconnect between iterations of a country’s NMSP. MPRs and mid-term reviews provide the most comprehensive evaluation of a programme’s processes and progress and have helped countries identify many of the challenges faced during implementation. These evaluations are only conducted during the middle and end of the NMSP cycle, and NMSPs have cited a low level of implementation of the MPR recommendations. These infrequent assessments result in programmes relying on their current SME methods, which often do not provide a complete assessment of their current progress toward targets.

The results showed a gap between previous NMSP accomplishments and current NMSP strategies. Without evidence-informed planning and decision-making, NMCPs struggle to achieve their targets, which are set to meet international guidelines. NMCPs may benefit from a revised NMSP development process to guide programmes in defining targets based on their country context and incorporate strategies to address previous challenges. The process should be informed by previous achievements. The development of better HIS systems, increased SME capabilities, and new modeling methods may assist programmes in developing a well-informed process and raise awareness of their gaps and capabilities. Guidance for setting realistic targets will create a relevant road map for programme planning and ensure continuous success, as countries work toward global malaria goals.

### Limitations of the study

This study was limited to the documents available through contacts at NMCPs and online searches. For some countries, the available population-based surveys were conducted within 1 to 2 years of the NMSP term. Although the survey did not perfectly align with the end of the NMSP, dramatic differences in coverage are not expected within this time frame. Out of the countries with a misaligned survey, Tanzania had an available subsequent survey (DHS 2015–2016), which was reviewed, and no targets were met. The type and timing of surveys varied across countries. The MIS is typically conducted during the rainy season compared to the DHS and MICS, which are conducted during the dry season. This variation makes it difficult to compare and assess trends over time. Policy changes may also affect the measurement of indicators and results and may affect trends observed. No further information was collected from key stakeholders involved in the NMSP development process.

## Conclusion

Sub-Saharan Africa has made significant strides in increasing coverage of malaria control interventions, which has helped reduce the overall malaria burden. To continue making progress toward the WHO Global Technical Strategy for Malaria and SDGs, it is critical that new NMSPs are informed by the accomplishments and challenges of previous iterations. This study revealed a disconnect between a country’s NMSPs, with most targets set according to internationally agreed-upon goals for Africa rather than an individual country’s accomplishments and capabilities. Implementation challenges were identified, but the solutions were not always fully incorporated into the new strategy. Guidance for programmes in setting realistic goals based on previous achievements and implementation challenges will allow countries to set and meet achievable targets as they work toward global goals.

## Additional file

**Additional file 1.** Extracted country challenges and solutions.

## Data Availability

The data that support the findings of this study are available from the Demographic and Health Surveys (DHS) Programme upon reasonable request and with permission of The DHS Programme and from the Multiple Indicator Cluster Survey. National malaria strategic plans may be found on each country’s NMCP website.

## Abbreviations

ANC: antenatal care
DHS: Demographic and Health Surveys
HIS: health information system
IPTp: intermittent preventive treatment in pregnancy
ITN: insecticide-treated net
IVM: integrated vector management
MICS: Multiple Indicator Cluster Survey
MIS: Malaria Indicator Survey
MoH: Ministry of Health
MPR: malaria programme review
NMCP: National Malaria Control Programme
NMSP: National Malaria Strategic Plan
RBM: Roll Back Malaria
SDG: Sustainable Development Goal
SBCC: social and behaviour change communication
SME: surveillance, monitoring, and evaluation
WHO: World Health Organization
